# Patterns of sexually transmitted infections in adolescents and youth in Dar es Salaam, Tanzania

**DOI:** 10.1186/1471-2334-6-22

**Published:** 2006-02-10

**Authors:** Guerino Chalamilla, Judica Mbwana, Fred Mhalu, Eunice Mmari, Mtebe Majigo, Andrew Swai, Willy Urassa, Eric Sandstrom

**Affiliations:** 1 Ilala Municipal Council, Dar es Salaam, Tanzania; 2 Muhimbili University College of Health Sciences, Dar es Salaam, Tanzania; 3 Karolinska Institute at Stockholm Soder Hospital, Stockholm, Sweden

## Abstract

**Background:**

Syndromic management of STIs has been advocated as simplified and cheap approach. Youth have been reported to be at increased risk of acquiring STIs which can facilitate HIV transmission. We have investigated the relationship between the syndromic management and specific aetiology diagnosis and its relationship with HIV infection and health seeking behaviour among youth attending a reproductive health clinic in Dar es Salaam, Tanzania.

**Methods:**

Between September 1998 and February 1999 among 1895 adolescents and youth below 25 years seen in the clinic 199 (10.5%) were randomly selected and consented to participate in the study. A standard questionnaire was administered. Blood and vaginal or urethral specimens were taken and investigated for STI causative agents.

**Results:**

Among a total of 199 studied adolescents and youth 22.6 % were teenagers, with fewer females 17.8% than males; 27.5% (p < 0.018). 20.8% of the females compared to 11.5% in males were HIV infected. Genital discharge was the most common complaint which was reported in 54.1% of male and 63.4 % of female patients. All males with gonorrhoea and four out of five with Chlamydia were given appropriate treatment with syndromic management, while 28% women with gonorrhoea or Chlamydia received appropriate treatment by syndromic management. All patients found with active syphilis by serology had not complained of genital ulcers and would not have been assigned to syndromic treatment for syphilis at the initial visit.

**Conclusion:**

The burden of STIs in this youth population is large indicating that youth are at increased risk of STIs and will certainly require youth friendly clinics. There is a need to refine the current syndromic management guidelines.

## Background

Sexually Transmitted Infections (STIs) especially genital ulcer diseases are an epidemiologic and biologic risk factor for the transmission of HIV-1 infection [1,2]. Young people aged 15 to 24 years are at the epicentre of the HIV epidemic especially those living in sub Saharan Africa which accounts for about 64.5% of people living with HIV/AIDS globally [3]. There are recent reports showing that adolescents in Africa engage in unsafe sexual behaviour which predisposes them to adverse outcome including sexually transmitted infections [4-7]. Consequently the youth in the continent have been reported to have high rates of various STIs [8-11].

Enhancing and optimising the management of STIs remains one of the most feasible and cost effective interventions to control them including HIV. A study done in Tanzania has shown that treatment of STIs can reduce HIV transmission by more than 40–60% [12]. The adoption of syndromic management has provided simplified and cheap method although its performance can be influenced by various factors including health seeking behaviour, cultural and economic factors, and the distribution of major aetiologies of STI syndromes [13].

We have investigated the relationship between the syndromic management and specific aetiology diagnosis in this group and its relationship with HIV infection and health seeking behaviour among youth attending a reproductive health clinic in Dar es Salaam.

## Patients and methods

The study was done between September 1998 and February 1999. The study population consisted of adolescents and youth below 25 years of age attending the youth reproductive health clinic. Recruitment of subjects was done randomly irrespective of having symptoms by blind selection of cards at the reception on arrival. Those who picked a yes card were invited and consented to participate in the study. A standardised questionnaire was administered to the recruited subjects and was used to collect details of socio-demographic information, sexual history, and basic knowledge on STIs and its prevention and on possible sources of the current sexually transmitted infection, history of present illness and its management. Answers were pre-coded with an exception of few which gave youth opportunity to answer open-ended questions.

All consenting youth were clinically examined including speculum examination in females. Free treatment was given to all those found with STIs using syndromic approach according to Tanzania national STI treatment guidelines. Sexual partners were invited to attend the clinic. Condoms, health education and counselling were offered to all. All were offered pre and post-test HIV counselling. Those who were found to be HIV infected, were offered further follow up counselling sessions, medical care and were referred to other institutions which provide different services for people living with HIV/AIDS.

### Laboratory methods

Syphilis antibodies were detected by the Venereal Disease Research Laboratory (VDRL – Murex Biotech Ltd, U.K) test and by Treponema Pallidum Particle Agglutination assay (TPPA) (Fujirebio Inc, Tokyo, Japan). Urethral swabs were taken from male patients while cervical and high vaginal swabs were taken from female patients. The swabs were inserted into Stuart transport media for culture of *Neisseria gonorrhoeae *and *Candida albicans*. Swabs for detection of *Chlamydia trachomatis *antigen were collected using appropriate collection kits. Using wet preparation all swab specimens were examined for the presence of pus cells, budding yeast cells and motile *Trichomonas vaginalis *in the referral laboratory. Smears from the urethral and cervical specimens were Gram stained and examined for the presence of typical bean shaped Gram-negative diplococci, budding yeast cells and clue cells. Clinically significant Candida infection was suggested by budding yeast cells in wet smears. Clue cells found on Gram stained smears were taken as an indication of bacterial vaginosis.

The swabs were inoculated onto modified Thayer Martin agar plates (Oxoid, Unipath Limited, Basingstoke, UK) supplemented with vancomycin, nystatin, colistin, and trimethoprim. The plates were incubated in a candle jar at 37°C for 48 hours. Presumptive identification of *N. gonorrhoeae *was done by Gram stain and oxidase test on suspected colonies. All isolates were confirmed to be *N. gonorrhoeae *by the Phadebact co-agglutination test (Phadebact, Monoclonal GC test, Boule Diagnostics AB, Huddinge, Sweden). Specimens were also inoculated onto Saboraud's Dextrose agar (Oxoid, Unipath Limited, Basingstoke, UK) and incubated at 37°C for 48 hours. Suspected colonies were identified as *C. albicans *using typical colonial morphology and germ tube test.

#### Antigen detection of *C. trachomatis*

Chlamydia infection was diagnosed using Chlamydia EIA MicroTrak II (Behring Diagnostics Inc. Cupertino, CA 95014, USA) following package insert instructions.

The presence of HIV antibodies was initially determined using Enzygnost anti-HIV-1 &2 Plus ELISA (Behring, Marburg, Germany) and sera found to be reactive were further tested using Wellcozyme HIV-1 recombinant ELISA (Murex, Dartford, UK) in an alternative confirmatory strategy for diagnosis of HIV infection [14]. Blood samples giving discordant results in the two assays were tested using Western blot assay (Genelab, USA), which was interpreted according to the World Health Organisation criteria for interpretation of Western blots [15].

### Statistical methods

Data were compiled and analysed by the SPSS 10.0 soft ware. Proportions were compared using chi-square (χ^2^) test or Fisher's exact test. A two tailed p-value <0.05 was considered significant.

## Results

Between September 1998 and February 1999, 1895 adolescents and youth below 25 years attended the youth reproductive health clinic at the Infectious Disease Centre (IDC). Among these, 199 participants consisting of 101 (50.8%) females and 98 (49.2%) males consented to participate in the study. Forty five (22.6%) were teenagers and 154 (77.4%) were youth aged 20 and 24 years. One third of females were already married compared to 9% of the males (Table 1).

**Table 1:** General characteristics of young people attending youth reproductive health clinic

Characteristic	Category	Males N (%)	Females N (%)
		98(49.2)	101(50.8)
Age group	15–19	27 (27.5)	18 (17.8)
	20–24	71 (72.5)	83 (82.2)
			
Marital status	Single	88(89.8)	64(63.4)
	Married	9(9.2)	32(31.7)
	Others	1(1.0)	1(4.0)
			
Sexual partners now	none	0	1 (1.1)
	1	54 (64.8)	84 (88.4)
	2–4	25 (31.6)	10 (10.5)
			
Sexual partners last 6 months	one	36 (40.0)	70 (73.7)
	2–4	52 (57.8)	25 (26.3)
	5–9	2 (2.2)	0
			
Sexual partners lifetime	one	6 (6.4)	24 (24.0)
	2–4	33 (35.5)	60 (60.0)
	5–9	34 (36.6)	14 (14.0)
	10 or more	20 (21.5)	2 (2.0)

### Sexual behaviour

The median age at coitarche was 17 years for both sexes. Among the females, 24% reported only one lifetime sexual partner, while 16% had five or more partners. Over the last 6 months the majority (73.7%) of females reported having only one sexual partner, and none had 5 or more sexual partners; 10.5% of females reported concurrent partners at the time of interview (Table 1). The mean duration of sexual activity among women was 3.4 years. Forty-six women reported 66 pregnancies while 22 had undergone 41 abortions (data not shown)

Among males, 6.4% reported one lifetime sexual partner while 58.1%, had five or more lifetime sexual partners. Over the last 6 months 60% reported having multiple partners and 31.6% acknowledged concurrent sexual partners at the time of interview (Table 1). The mean duration of sexual activity was 4.5 years. Among the participants all except 5 acknowledged having practised vaginal-penile sex while 11% and 7% of the males; 7% and 16% of the females had practiced oral and anal sex, respectively.

### Comparison of the syndromic and aetiological diagnosis of patients

Genital discharge was the most common complaint that was reported by 54.1% of male patients and 63.4 % of female patients. Out of 199 patients recruited into the study, culture was done on 110 (55.2%) patients who did not respond after seven days of treatment using syndromic management. These included 28/98 (28.6%) male and 82/101(81.2%) female patients. There was no significant differences on the sexual characteristics including the mean age at coitache, the mean number of lifetime sex partners or mean years of sexual activity among those who had culture done compared to those who had no culture done. *N. gonorrhoea *was isolated in 13 (54.1%) of 24 male samples, but in only 2 (3.6%) of 55 female samples. *C. trachomatis *antigen was detected in 9.7% in the males and 3% in women. Six out of seven patients who were found to have active syphilis using serology were found to have other infections including *N. gonorrhoea*, candidiasis, trichomoniasis and bacterial vaginosis. Among women complaining of genital discharge candidiasis was found in 26.6%; bacterial vaginosis in 16.9% while trichomoniasis was found in 10.9% (Table 2).

**Table 2:** Aetiological diagnosis versus presenting symptoms of sexually transmitted diseases among patients.

Aetiological diagnosis			Presenting symptoms									
	Total		Genital discharge		Genital ulcers		Dysuria		Genital itch		Rash	

	M No (%)	F No (%)	M No (%)	F No (%)	M No (%)	F No (%)	M No (%)	F No (%)	M No (%)	F No (%)	M No (%)	F No (%)

			53/98 (54.1)	64/101 (63.4)	14/98 (14.3)	12/101 (11.8)	34/98 (34.7)	12/101 (11.8)	9/98 (9.1)	31/101 (30.6)	7/98 (7.1)	14/101 (13.8)
Gonorrhoea	14/28 (50.0)	3/82 (3.7)	13/24 (54.1)	2/55 (3.6)	0/1	1/7	1/4	0/10	0/2	2/28 (7.1)	1/1	1/9
Chlamydia	5/52 (9.6)	5/56 (8.9)	3/31 (9.7)	1/37 (3)	1/6	0/5	1/17	1/7	1/3	2/10	0/2	2/4
Syphilis	3/98 (3.1)	4/101 (4.0)	2/53 (3.8)	4/60 (6.7)	0/14	0/12	0/34	0/12	0/9	1/31	0/7	1/14
Clue cells	-	15/88 (17.0)	-	10/59 (16.9)	-	1/8	-	3/11 (17)	-	8/31 (25.8)	-	3/8
Trichom onas	2/94 (2.1)	10/101 (9.9)	2/53 (3.8)	7/64 (10.9)	0/11	0/12	0/33	1/12	0/9	5/31 (16.1)	0/7	2/14
Candida	1/97	22/101 (21.8)	1/44	17/64 (26.6)	1/13	1/12	2/34	4/12	0/9	7/31 (22.6)	0/7	3/11
HIV	11/96 (11.5)	21/101 (20.8)	6/53 (11.3)	12/64 (18.8)	3/13 (23.1)	6/12 (50.0)	2/33	1/12 (8)	1/8	6/31 (19.3)	1/7	2/14

**Table 3:** HIV serostatus in relation to syndromic or aetiological diagnosis of various STIs

	HIV- seropositive		HIV- seronegative	
STI agent/syndrome	Males (n = 11) No (%)	Female(n = 21) No (%)	Males (n = 85) NO (%)	Females (n = 80) No (%)

Genital discharge	7/11 (63.6)	15/21 (71.4)	67/85 (78.8)	72/80 (90.0)
Genital ulcer	3/11 (27.2)	9/21 (48.9)	16/85 (18.8)	18/80 (22.5)
				
N. gonorrhoeae	3/4(75.0)	0/15	11/24 (45.8)	3/67 (4.5)
C. trachomatis	1/3 (33.3)	2/13 (15.4)	4/48 (8.3)	3/40 (7.5)
Bacterial vaginosis	-	3/17 (17.6)	-	12/71 (16.9)
T. vaginalis	1/11 (9.1)	1/21 (4.8)	1/81 (12.3)	9/80 (11.2)
C. albicans	0/11	1/20 (5.0)	3/82 (3.6)	21/80 (26.2)
Syphilis	1/11 (9.1)	2/21 (9.5)	2/85 (2.3)	2/78 (2.6)

All males who were found to have gonorrhoea and four out of five with Chlamydia received appropriate treatment based on syndromic management approach. This is in contrast to only two out of seven women who were found to have either gonococcal or chlamydial infections who were appropriately treated syndromically.

A genital ulcer was the reason for attendance for 14.3% of the males and 11.8% of the females and was the clinical diagnosis in all with exception of one in each sex. Genital ulcer as a guide to initiate treatment for syphilis was clearly inadequate since none of the seven patients with active syphilis using serology were among the 26 who complained of genital ulcer. Among these seven patients one was found to have gonorrhoea, two had candidiasis, one had trichomoniasis and two had clue cells suggestive of bacterial vaginosis. Among the seven patients there were no clinically suspected cases of chanchroid or Lymphogranuloma venerium. A specific diagnosis for Herpes genitalis infection was not available during the study.

Two males presented with both genital discharge and genital ulcer compared to 12 females (Table 2). Only three of the women with signs suggestive of bacterial vaginosis, trichomoniasis or yeast infection had multiple agents. Three of the 6 females with a provisional diagnosis of pelvic inflammatory disease (PID) had an Enzyme Immunoassay for Chlamydia done and all were negative. One of the 3 women with gonorrhoea was diagnosed with PID. Among 16 women who used intra uterine devices one had PID.

### HIV in relation to behavioural factors

Among all the participants, 20.8% of the females were HIV infected, compared to 11.5% in males. Among the 45 teenagers; 3/27 (11.1%) males and 3/18 (16.6%) females were found to be HIV-1 infected. In males the risk of being HIV infected was relatively constant with regard to number of partners up to 10 lifetime partners. However when the number of lifetime sexual partners was more than 10, the risk of HIV infection rose to 26.3%. In females an increased risk to be HIV infected was already seen in those with more than 4 lifetime sexual partners and the risk by then rose to 43.8%. The current number of partners or partners over the last 6 months was not associated with risk of being HIV infected, although it may have influence the risk of transmitting the virus.

Out of 22 women with history of abortions 6 (27.3%) were found to be HIV infected and of the 26 women who had given birth to live children 5 (19.2%) were HIV infected. Of the 9 married males 2 (22%) were HIV infected compared to 7/32 (21.9%) married females. The rate of HIV infection among those who were not married was 10.5% and 18.8% for males and females respectively. Among women who acknowledged having only one lifetime partner, 4/24 (17%) were HIV infected, three of whom were married.

Most of the sexual partners, at the intercourse when the current STI was thought to have occurred, were single. The most common meeting place was at home 94/120 (78%) and majority of these sexual partners were considered as friends 96/127(76%). However, it was also common that the sexual partners were married and most of these encounters were also at home 40/45 (89%). Though the questionnaire did not define whether some of them were their spouses, 28/36 (78%) defined them as a 'friend'. Females 38, more often had married partners than males, 8, p < 0.001. Seven (18%) of the females whose last partner was married, were HIV infected.

### HIV in relation to syndromic and specific diagnosis

There was a non-significant trend to less GDS in HIV infected compared to non-infected in males (64 vs. 79%) and females (71 vs. 90%) respectively, which was significant if the genders were combined, p = 0.045. Regarding GUD there was a non-significant trend to more GUD in HIV infected compared to non-infected in males (27 vs. 19%) and females (49 vs. 23%) respectively, which remained non-significant even if the genders were combined. The small numbers preclude a comparison between HIV serostatus and specific diagnosis, however it was noted that 3 of 4 males with gonorrhoea were HIV infected.

### Condoms use

81 males (82.6%) and 94 females (93.1%) reported to have ever used a condom. There was no association between condom use and HIV status, current sexual activity, number of lifetime sexual partners or duration of sexual activity. About 10% of males and 6% of females had used a condom the day the current STI was thought to have occurred. Eighty percent of males and females knew that condoms could protect against STIs. There was no relationship between this knowledge and HIV serostatus.

### Health seeking behaviour

The median number of days with STI symptoms was 14 days with a wide range (1–720) for men and 19 days with a range (3–720) for females (Table 4). Thus it seems that the clinic is readily available for a subpopulation, while others are greatly underserved. Gonorrhoea, chlamydia, syphilis, genital discharge or rash, were associated with the shortest delays in men, while trichomoniasis, dysuria and genital discharge caused the shortest delays in women. However, genital ulcers were not associated with short delays. HIV serostatus did not seem to affect the health seeking behaviour, at least in men.

**Table 4:** Median days of symptoms before visit to the clinic according to symptoms or aetiological diagnosis

	Males		Females	
	Median (range)	N	Median (range)	N

Genital Discharge	8.5 (1–720)	50	14 (3–720)	59
Genital ulcer	14 (14–120)	13	30 (14–180)	12
Dysuria	21 (3–720)	34	10.5 (3–360)	12
Genital itching	25.5 (7–120)	8	45 (3–360)	30
Genital rash	8.5 (3–14)	6	30.5 (5–330)	14
Genital swelling	14	1	30 (7–120)	3
				
Gonorrhoea	3 (1–10)	13	60 (7–90)	2
Chlamydia	7 (3–21)	5	31 (3–240)	5
Syphilis	8.5 (3–14)	2	60 (3–180)	2
Trichomonas	62 (4–120)	2	14 (7–60)	10
Candida	-	-	20 (3–360)	9
BV	-	-	31 (3–240)	13
HIV	12.5 (3–150)	10	25.5 (3–180)	20
				
Any	14 (1–720)		19 (3–720)	

Factors associated with disempowerment were associated with staying for longer periods with symptoms before seeking medical attention. Four males and nine females, who had not consented to the last intercourse, waited a median of 90 and 60 days respectively. Fourteen women could not advice their sexual partner to come while eight agreed to be tested but did not wish to know their HIV serostatus. These two groups of women were associated with median delays of 45 and 45 days respectively. These observations form a pattern but are not individually statistically significant due to the great variance. Those 33 females who could persuade their male partners to come to the clinic had a shorter median delay of 14 days, p = 0.018. None of the other sociological, sexological or clinical factors seemed to influence the delay to seek medical care. Most had heard about syphilis and gonorrhoea, however only two thirds regarded HIV as an STI.

### Referral

Ninety four percent of young people stated that they attended the clinic due to symptoms; however 16 women were notified by their partners to attend, of whom 12 were asymptomatic. Four of the 16 females who were referred by their sexual partners were HIV positive. However, at the end of the study, 16 female partners to study males and 33 male partners to study females had been investigated at the IDC outside the study and a further 8 patients were treated elsewhere, in addition 5 patients were identified as index cases outside the study. Thus the counselling initiated by the study dramatically changed the referral pattern from a minority of females who had heard of the disease from their partners to a referral of male partners of almost one third of the affected females.

## Discussion

We have previously shown the high rate of STIs among youth in Dar es Salaam,[9] and have now proceeded to further study possible avenues of intervention. In order to treat STIs it is not only important that health care can be sought when symptoms appear, but also that the subsequent treatment cures the disease and prevents re-infections. This requires knowledge on the part of youth and availability of health care facilities as well as appropriate diagnosis, treatment and referral procedures. The clinic has continued to attract a large number of youth primarily through the word of mouth and the awareness of STIs is generally widespread in this population, although only two thirds had heard that HIV was spread as a STI. Although studies like this one relies on self-reported data it is worth noticing that an illegal procedure such as abortion was acknowledged by 21.8% of women, which can be taken as a sign of the atmosphere in the clinic. However inconsistencies do occur such as 7 men and 3 women reporting a younger current age than when they commenced sexual activity.

All but 12 patients reported STI symptoms as the reason for attendance. It remains to be seen if the increase in asymptomatic patients since the last investigation is a sign of a trend towards greater acceptability of the clinic. The most common complaint in both men and women was genital discharge. In men, gonorrhoea dominated with 50%, followed by chlamydia (10%) and trichomoniasis (4%). In women vaginal candidiasis (27%), bacterial vaginosis (17%) and trichomoniasis (11%) dominated while gonorrhoea (3.6%) and chlamydia (3%) were relatively rare causes of genital discharge.

These figures should be viewed with some caution and probably underestimates the importance of the various agents. Culture for gonococci followed standard techniques and the transport was adequate, but gonococci are notoriously sensitive. The relatively low proportion of males with genital discharge and gonorrhoea in this series could reflect the relative lower sensitivity of culture in diagnosis of *N. gonorrhoeae *infection as reported elsewhere[16,17]. Chlamydia was detected by ELISA which is not an optimal technique [17], but the rate among men is approximately that expected from studies in industrialized countries, while the low frequency in women has been found in previous studies from Dar es Salaam [9] and could be due to lack of sensitivity in the test or a predominance of other causes of discharge. Clue cells on Gram stains taken from swabs transported to the laboratory were taken as an indication of bacterial vaginosis and are a reasonable approximation of bedside performed pH, sniff test and wet mounts [18]. Viable trichomonads was sought on saline diluted swabs in the referral laboratory and probably underestimates its importance, while the notoriously difficult laboratory diagnosis of relevant Candida infection was estimated from the presence of budding yeast cells in wet smears. Of the three men who had positive smears, culture yielded one isolate of *C. albicans *while the remaining two isolates were other Candida species. Of the 22 women patients with positive smears only one had a positive culture due to *C. albicans *while 6 were other Candida species. Patients with sera giving positive VDRL and TPPA tests were considered to have active syphilis. An additional 7 males and 3 females had only a positive TPPA indicating past syphilis.

Syndromic treatment was appropriate in men with GDS since gonorrhoea and chlamydia dominated. However GDS in females can be caused by many agents that include gonococci, Chlamydia, anaerobic bacteria, *T. vaginalis *and Candida. As a results most of diagnostic assays perform better in males compared to females as reported elsewhere [16,17]. In that regard syndromic management in women with GDS may be a better approach than using laboratory results.

In this clinic, herpes simplex as in other studies has been found to be the major cause of genital ulcers (>60%) and chancroid to be rare (<10%). The use of syndromic algorithm for GUD to diagnose and treat syphilis was found to be very inappropriate since none of those with ulcer had active syphilis, rather it was associated with agents causing genital discharge. The study justifies suggestion of the inclusion of RPR test in syndromic protocols that could provide appropriate therapeutic cover for syphilis as syphilis control is a priority within STI control programs. There were fewer tendencies to be HIV infected in those with genital discharge but a trend to be more likely to be HIV infected in those with genital ulcers.

HIV infection was found in 21% of females and 12% of males which is slightly lower for females and higher for males than in the previous study [9]. These high figures are found without a particularly high-risk behaviour among the females, although their vulnerability is illustrated by the high number of unwanted pregnancies. One quarter had so far adhered to the public health advice of having only one partner, this did not protect against HIV infection nor did marriage. Very few had however used a condom with the partner at the intercourse they thought was the cause of the current STI even though it was often with a friend and occurred in the relatively safe setting of the home.

Teenagers seem to be exposed to a similar risk for STIs as older youth and services should be targeted even more to their needs. Teenagers did not differ in their duration with symptoms prior to the visit to the clinic. It seemed that factors that could be taken as disempowerment such as involuntary intercourse, inability to advice sexual partner to come, not wanting to know their HIV serostatus were associated with delays, while women who thought they could persuade their partners to attend had a shorter delay. The great span in delays was striking. Some obviously had a very low barrier to seek care while others had the symptoms for long periods of time, with the risk of further transmission and risk for complications.

Partner referral has generally been considered to be very difficult in developing countries, especially when only syndromic treatment is available. The large number of causes of female GDS are not necessarily a rationale for the male partners to go for treatment of gonorrhoea or chlamydia, while females partners to men with GDS have good reason to attend even if asymptomatic. It is therefore interesting to note that 16 women attended this study due to notification from their partner and that the counselling during the study caused a further 16 females to attend outside the study and may be more remarkably 33 males to female study partners attended the clinic outside the study. Thus there seems to be a great potential for counselling in the specific setting of a youth clinic.

However although males and females attended the same clinic it is an oversimplification to assume that in general they have sexual relationship. We have found in this study, as previously reported that males reported more current, recent and lifetime partners than females. In addition men had 0.14 (SD3.0) and 2.15(SD 3.18) years younger partners at the first and last intercourse respectively, the corresponding figures for females were 4.66(SD2.31) and 6.77 (SD 2.54) years older partners (data not shown). Thus these females in general seem to have most of their partners outside the age span in which we are recruiting men and a different sexual behaviour. It is thus a challenge to identify the sexual partners to the young men we recruit in these studies.

## Conclusion

The burden of STIs in this youth population is large indicating that youth are at increased risk of STIs and will certainly require youth friendly clinics. There is a need to refine the current syndromic management.

## Competing interests

Authors have no competing interest regarding this work which we are submitting for publication.

## Authors' contributions

GC participated in the supervision of the study and writing of the manuscript. JM supervised testing of the samples in the reference laboratory and manuscript writing. WU participated in statistical analysis and manuscript writing. FM participated in the design, supervision of the study and manuscript writing. EM participated in conduct of the study. MM participated in manuscript wring. AS participated in the supervision of data entry and statistical analysis, ES participated in the design, statistical analysis and manuscript writing. All authors read and approved the final manuscript.

## Pre-publication history

The pre-publication history for this paper can be accessed here:


